# Barriers to epilepsy care: Measuring the treatment gap in rural and peri-urban districts in Ghana

**DOI:** 10.1371/journal.pone.0355854

**Published:** 2026-08-11

**Authors:** Emmanuel Kwame Darkwa, Patrick Adjei, Sabina Asiamah, Elizabeth Awini, Cynthia Sottie, Anthony Godi, John E. Williams, Frank Atuguba, Fuseini Sataru, Alexander Adjei, Arjune Sen, Charles R. Newton, Anthony Danso-Appiah, J. Helen Cross, Josemir W. Sander, Albert Akpalu, on behalf of the EPInA investigators

**Affiliations:** 1 Dodowa Health Research Centre, Research and Development Division, Accra, Ghana; 2 Ghana Health Service, Accra, Ghana; 3 Department of Epidemiology and Disease Control, School of Public Health, University of Ghana, Legon, Accra, Ghana; 4 University of Ghana Medical School, Accra, Ghana; 5 Korle Bu Teaching Hospital, Korle Bu, Accra, Ghana; 6 Department of Biostatistics, School of Public Health, University of Ghana, Legon, Accra, Ghana; 7 Oxford Epilepsy Research Group, Nuffield Department of Clinical Neurosciences, John Radcliffe Hospital, Oxford, United Kingdom; 8 Department of Psychiatry, University of Oxford, United Kingdom; 9 Centre for Evidence Synthesis and Policy, University of Ghana, Legon, Accra, Ghana; 10 UCL Great Ormond Street NIHR BRC Institute of Child Health, London, United Kingdom; 11 UCL Queen Square Institute of Neurology, London, United Kingdom; 12 Department of Neurology, West China Hospital, Sichuan University, Chengdu, China; Niigata University of Health and Welfare: Niigata Iryo Fukushi Daigaku, JAPAN

## Abstract

**Background:**

Epilepsy is an easily treatable condition, but a substantial treatment gap persists, particularly in low- and middle-income countries. We quantified the epilepsy treatment gap in Ghana.

**Materials and methods:**

We conducted a three-stage population-based study in the Shai Osudoku and Ningo-Prampram districts, embedded within the Dodowa Health and Demographic Surveillance System to estimate prevalence and assess treatment barriers from April 2023 to December 2023.

**Results:**

We found an epilepsy prevalence of 8.84 per 1,000 people (95% CI: 8.00–9.68). A 70% treatment gap was identified, with only 30% of those with a confirmed diagnosis using antiseizure medication. Among untreated people, the most common reason was a lack of diagnostic awareness (37.8%). Barriers to treatment included sociocultural beliefs and misconceptions, stigma, preference for alternative medicine, cost, and antiseizure medication shortages. Significant knowledge gaps were evident, with 36.8% of respondents attributing epilepsy to witchcraft or supernatural causes.

**Conclusion:**

Most people with epilepsy in the area were not receiving adequate biomedical care. The observed treatment gap was associated with multiple factors, including gaps in awareness, sociocultural misconceptions, and economic barriers, suggesting the need for targeted public health interventions to improve epilepsy care and management.

## Background

Epilepsy is a manageable condition [1], imposing a significant multidimensional burden worldwide [2–4]. In low- and middle-income countries (LMIC), where 80% of the over 50 million affected people live, the substantial burden is underpinned by a wide treatment gap [2–5]. Most people with epilepsy can achieve seizure freedom and experience improved quality of life with access to first-line antiseizure medications (ASM) costing less than US$10 annually [1,6].

Disparities in epilepsy treatment rates across and within countries mainly result from unequal access to healthcare [7–9]. In resource-limited settings, systemic barriers weaken diagnostic capacity and access to care, increasing the risk of untreated epilepsy. These barriers include disparities in geographic access, fewer specialists, and a lack of advanced diagnostic technology [10–12]. In sub-Saharan Africa (SSA), particularly, these systemic weaknesses are amplified by entrenched cultural misconceptions, as well as the cost and availability of ASM [13–17].

Similar to other LMICs, Ghana has a large epilepsy treatment gap, with most people with epilepsy lacking access to biomedical care [1,6]. Critical knowledge gaps exist regarding the extent and contributing factors nationwide, complicating a nationwide understanding of the issue, enabling targeted interventions, and achieving better outcomes. Quantifying these gaps is crucial to improving treatment rates and outcomes and to developing targeted interventions. We aim to identify barriers and quantify the local epilepsy treatment gap in two districts of Ghana using a comprehensive, three-stage prevalence survey. This study is part of a broader project generating current epidemiological data on epilepsy in southeastern Ghana.

## Methods

### Setting

**Fig 1:**
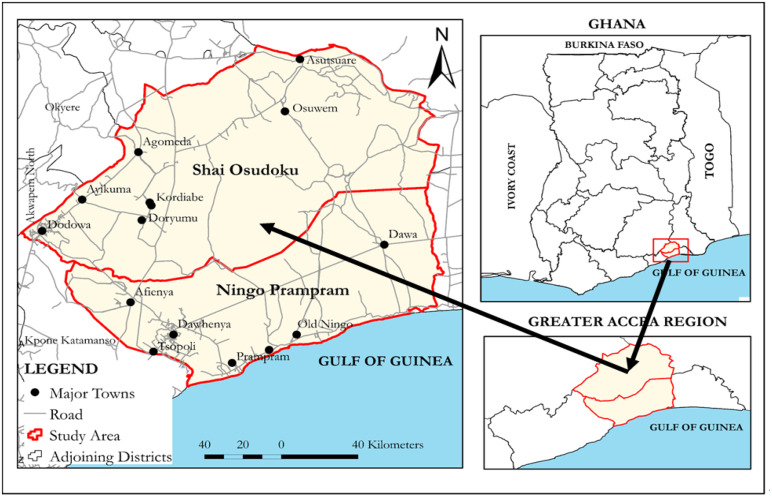
Map of Ghana highlighting the two study districts of Shai-Osudoku and Ningo-Prampram within the Greater Accra Region, including major towns, roads, and neighboring districts. Adapted from [22].

The study was conducted in the Shai-Osudoku and Ningo-Prampram districts, located in the Greater Accra region and bordering each other along Ghana’s southeastern coast (see Fig 1) [18]. They share several characteristics, including a mix of Ga-Dangme, Ewe, and Akan ethnic groups. Shai-Osudoku is mainly rural and agrarian. It contains a network of health facilities, including a modern district referral hospital, three health centres, eight Community-Based Health Planning Services (CHPS) compounds, and four private facilities. In contrast, Ningo-Prampram is a peri-urban area with a more diverse economy, including one polyclinic, three health centres, ten CHPS, and twenty-six private facilities, located along the coast. Both districts are located within the boundaries of the Dodowa Health and Demographic Surveillance Site (DHDSS), which monitors population and health trends and serves as a sampling frame for research and interventions [19–21].

### Study design

We conducted a community cross-sectional survey using a door-to-door data collection approach, including a matched case-control study. Using the DHDSS database, we enumerated long-term residents to quantify the prevalence of epilepsy and the treatment gap. This work is a component of the Epilepsy Pathway Innovation in Africa (EPInA) project (https://epina.web.ox.ac.uk/home). It aims to improve the quality of life for people with epilepsy in SSA by addressing all aspects of the treatment pathway: prevention, diagnosis, treatment, and awareness. The project began on 1 October, 2019 and ended on 31 January, 2025.

### Participant recruitment and screening

We undertook significant preparatory work before conducting the door-to-door survey. The initial step was to develop and validate a method for case identification. The team reviewed existing tools to create a contextually relevant 17-item screening tool. After translating it into the local languages, we tested and confirmed its effectiveness at multiple health facilities across the districts and a major referral hospital. In a formal validation, the tool was administered to 70 people with epilepsy and 70 controls in Dangme, and to 50 people with epilepsy and 50 controls in Asante Twi [23]. The screening tool demonstrated acceptable sensitivity and specificity for population-based identification of epilepsy. Full validation results are reported elsewhere [23].

Concurrently, the team worked to reduce stigma associated with epilepsy and secure community acceptance by consulting with local stakeholders. Subsequently, research assistants with community experience underwent a 10-day neurologist-led training to standardize knowledge of seizures, terminology, and questionnaire administration using role-play and instructional videos. The training ensured that the screening questions were administered consistently and uniformly. We determined the prevalence of epilepsy through a systematic three-stage door-to-door survey. The three-stage approach consisted of population screening, detailed individual assessment, and clinical confirmation by specialists.

Three contact attempts were made before classifying households as unavailable. The protocol involved a progressive screening process, culminating in a clinical evaluation. Stage 1 involved a broad screening process using a 7-item checklist, in which a single positive response constituted a potential case. Stage 2 used a 10-item questionnaire. Lastly, clinical confirmation, supported by a physician or neurologist’s assessment and an electroencephalogram (EEG), was used to determine the type of epilepsy. We operationalized “lifetime epilepsy” as confirmed epilepsy at any time before the survey, and “active epilepsy” as treatment or seizures within the past year [10,24].

We used a census approach within the DHDSS to cover eligible residents in the Shai-Osudoku and Ningo-Prampram surveillance areas. The combined DHDSS population of 136,727 served as the sampling frame. The flow of participant recruitment and the three-stage screening process have been reported elsewhere [22].

Following identification, we compared people with confirmed active epilepsy (“cases”) to people without epilepsy (“controls”). We entered an algorithm into the DHDSS database that identified 421 cases and accurately matched them to controls by age and sex. As there were many potential controls, the matching was random. Eventually, the groups consisted of 366 people with epilepsy and 312 controls.

### Data collection

Android tablets running the KoboCollect app (version 2023.2.4) were used to collect data. In addition to the socio-demographic and economic information collected from participants, a modified questionnaire adapted from a previous study [25] was used to compile a comprehensive clinical profile for cases. The information collected included seizure details, risk factors, lifestyle factors, treatment history, and barriers to accessing care. Age-specific questionnaires were administered, with caregivers responding on behalf of children or individuals with cognitive impairments. Cases were interviewed at health facilities, and controls at their homes.

### Definitions

Epilepsy treatment gap: Treatment status was determined by self-report of any ASM use within the 30 days preceding the survey, irrespective of dose, adherence, or seizure control. The treatment gap was estimated as the percentage of those with active epilepsy who did not report ASM use during this period. This operational definition captures treatment access, although it may not fully distinguish between untreated epilepsy, undertreatment, interrupted treatment, or poor adherence; these nuances should be considered when interpreting the estimates.

**Prevalence of epilepsy** is the proportion of individuals in a defined population who have epilepsy at a specified point in time or period.

**Treatment barriers** are factors that limit access to, initiation of, or continuity of appropriate epilepsy care, including individual, family, health system, economic, cultural, and geographic influences.

**The diagnostic gap** refers to the proportion of people with epilepsy who have not been correctly identified or diagnosed by the health system.

**The Therapeutic gap** refers to the proportion of people with epilepsy who are diagnosed but do not receive effective and sustained treatment.

**Treatment adherence** refers to the extent to which a person with epilepsy takes antiepileptic medication as prescribed in terms of dose, frequency, and duration.

**Appropriate treatment** refers to evidence-based antiepileptic therapy prescribed at an adequate dose and duration, tailored to seizure type, syndrome, comorbidities, and local guidelines.

**Active epilepsy:** a history of epilepsy with at least one seizure in the past 12 months and/or current use of antiseizure medication

### Statistical analysis

We processed and analyzed the data using a predefined statistical workflow. Data were cleaned in KoboToolbox and Microsoft Excel, and analyses were conducted in Stata(Stata/SE 17; StataCorp, College Station, TX, USA). Clinical characteristics, treatment history, and perceived barriers were summarized using descriptive statistics and reported as frequencies and percentages. Comparisons between districts were performed using Pearson’s chi-square test for categorical variables. Fisher’s exact test was applied where expected cell counts were small (<5). We estimated the crude prevalence of epilepsy by dividing the number of clinically confirmed cases by the total number of individuals screened. We used inverse probability weighting to adjust for attrition bias. Weights were derived from logistic regression models predicting stage-to-stage participation based on DHDSS demographic variables. The weights accounted for the probability of completing each screening stage, with covariates including age, sex, district, and screening-stage positivity. To account for potential household and community-level clustering inherent in the door-to-door sampling design, cluster-robust standard errors were estimated for all comparative analyses. Potential confounding by age and sex was controlled via exact matching during the case-control selection phase. The treatment gap was estimated as the proportion of active epilepsy cases that did not receive appropriate treatment [5,7] based on the operational definition of treatment status. We estimated p-values and set statistical significance at *p < 0.05*.

### Ethical considerations

Ethical approval was obtained from the Ghana Health Service Ethics Review Committee (GHS-ERC 022/08/20) and the Dodowa Health Research Centre Institutional Review Board (DHRCIRB 95/08/20. Participants provided written informed consent. Community consent was secured through local leaders. Written informed consent was obtained from adults. For minors, parents provided assent. Interviews were conducted in the preferred languages (Dangme, Twi, and English) with interpreters as needed. Data were anonymized pre-analysis. Those with epilepsy were registered for continuous clinical follow-up.

## Results

### Prevalence of epilepsy

The initial screening covered 65,177 people across both districts, of whom 617 were identified as suspected cases at stage 1 (Table 1). The prevalence of suspected epilepsy at Stage 1 varied significantly between districts, with a higher proportion observed in Shai-Osudoku (1.3%) than in Ningo-Prampram (0.6%) (p < 0.001). During Stage 2 screening, 544 individuals were evaluated, of whom 467 were suspected of having epilepsy. Of the 435 individuals who underwent clinical assessment, 421 were confirmed to have epilepsy. However, no significant district differences were observed at Stage 2 screening (85.3% vs 86.8%; p = 0.634) or in confirmed epilepsy cases following clinical assessment (96.6% vs 97.1%; p = 0.766). Not all attrition in screening stage transitions reflected loss to follow-up. Of the 544 participants assessed at Stage 2, 72 (13.2%) screened negative and were excluded from Stage 3 clinical assessment as false positives from the initial broad screening tool, consistent with the multi-stage design. Overall, the attrition-adjusted prevalence of epilepsy was 8.84 cases per 1,000 people.

**Table 1:** Three-stage screening for epilepsy.

	District, N (%)			
	Ningo-Prampram	Shai-Osudoku	Total	P-value*
Stage 1 screening	36,965	28,212	65,177	
Suspected cases	237 (0.6)	380 (1.3)	617 (0.9)	<0.001
Stage 2 screening	204	340	544	
Suspected cases	177 (86.8)	290 (85.3)	467 (85.8)	0.634
Stage 3 screening	176	285	461	
Suspected cases	172 (97.7)	263 (92.3)	435 (94.4)	0.014
Confirmed cases within those still suspected	167 (97.1)	254 (96.6)	421 (96.8)	0.766

* P-values were derived using Pearson’s chi-square test for categorical variables. Fisher’s exact test was applied where expected cell counts were <5.

### Epilepsy treatment coverage and gap

The epilepsy treatment gap was 70.3% as only 29.7% of people with confirmed epilepsy were taking ASM in the month before the survey (Table 2). A marked difference in ASM uptake was observed between the districts, with a higher rate in Ningo-Prampram (35.3%) than in Shai-Osudoku (26%). However, this did not differ significantly between the districts (p = 0.183). Most (93.6%) of those on treatment were receiving prescriptions from physicians or nurses. Similarly, no statistically significant district-level differences were observed in ASM prescribers (*p* = 0.387). The most common ASMs used were Carbamazepine (66.4%) and Phenobarbital (19.2%). A significant proportion of cases (11.2%) did not know the names of their medications, and this was markedly higher in Shai-Osudoku (16.6%) than in Ningo-Prampram (5.1%). ASM effectiveness was good, with a 72% reduction in seizure frequency, and an additional 10% of people reported being seizure-free since starting treatment. However, 11% reported their medication was ineffective. Adherence was a significant challenge, with 43.2% of participants missing doses in the past month, with higher nonadherence rates in Shai-Osudoku (45.5%).

**Table 2:** Treatment history and associated factors of epilepsy.

	District, N (%)			
	Ningo-Prampram	Shai-Osudoku	Total	P-value**
Amongst confirmed cases,	167	254	421	
Those on treatment for at least the past 1 month	59 (35.3)	66 (26.0)	125(29.7)	0.183
For those on treatment,				
Prescribers of the medication*				
Doctor/Nurse	56 (94.9)	61 (92.4)	117(93.6)	0.387
Relative/Friend	2 (3.4)	3 (4.5)	5 (4.0)	
Pharmacy attendant/Drug store owner	1 (1.7)	0 (0.0)	1 (0.8)	
Other	0 (0.0)	2 (3.0)	2 (1.6)	
Types of ASM				
Carbamazepine	39 (66.1)	44 (66.7)	83 (66.4)	
Phenobarbital	14 (23.7)	10 (15.2)	24 (19.2)	
Sodium Valproate	2(3.4)	1(1.5)	3 (2.4)	
Phenytoin	1(1.7)	0 (0.0)	1 (0.8)	
Others (Unknown)	3(5.1)	11(16.6)	14 (11.2)	
Effect of this medication on seizures				
Seizures have stopped	8 (13.6)	5 (7.6)	13 (10.4)	0.579
Seizures have reduced	42 (71.2)	49 (74.2)	91 (72.8)	
No effect	6 (10.2)	8 (12.1)	14 (11.2)	
Seizures have got worse	0 (0.0)	2 (3.0)	2 (1.6)	
Don’t know	3 (5.1)	2 (3.0)	5 (4.0)	
Sources of ASM*				
Pharmacy	53 (89.8)	59 (89.4)	112 89.6)	0.487
Roadside drug store/Mobile drug vendor	0 (0.0)	1 (1.5)	1 (0.8)	
Donation from family/well-wishers	1 (1.7)	3 (4.5)	4 (3.2)	
Other	5 (8.5)	3 (4.5)	8 (6.4)	
Missed taking medication within the past 1 month	24 (40.7)	30 (45.5)	54 (43.2)	0.59
For those not on treatment,	108	188	296	
Reasons for not being on treatment*				
Not aware they have epilepsy	19 (17.6)	93 (49.5)	112(37.8)	<0.001
Don’t think ASM are effective	25 (23.1)	37 (19.7)	62 (20.9)	
ASM are too costly	30 (27.8)	36 (19.1)	66 (22.3)	
ASM are not available in pharmacy	3 (2.8)	2 (1.1)	5 (1.7)	
Fear of side-effects	3 (2.8)	1 (0.5)	4 (1.4)	
Other	45 (41.7)	35 (18.6)	80 (27.0)	
Within confirmed cases,	167	254	421	
Ever taken traditional medicine for epilepsy	108 (64.7)	177(69.7)	285(67.7)	0.282
Ever consulted a priest/pastor/man of God for prayers or deliverance from epilepsy	138 (82.6)	179(70.5)	317(75.3)	0.005
Perceive overall management of their epilepsy as adequate	31 (18.6)	35 (13.8)	66 (15.7)	0.187
Perceived causes of their epilepsy*				
Family/genetic trait	5 (3.0)	14 (5.5)	19 (4.5)	0.082
Illness	37 (22.2)	41 (16.1)	78 (18.5)	
Previous injury	6 (3.6)	4 (1.6)	10 (2.4)	
Spirits/curses	65 (38.9)	90 (35.4)	155(36.8)	
Don’t know	61 (36.5)	106 41.7)	167(39.7)	
Other	14 (8.4)	11 (4.3)	25 (5.9)	
Person/caregiver believes that they can be cured of epilepsy				
No	10 (6.0)	5 (2.0)	15 (3.6)	0.016
Yes	129 (77.2)	221(87.0)	350(83.1)	
Don’t know	28 (16.8)	28 (11.0)	56 (13.3)	
For those who believe they can be cured				
What they think is the best option for them to be cured				
Medication	83 (64.3)	139(62.9)	222(63.4)	0.632
Surgery	1 (0.8)	1 (0.5)	2 (0.6)	
Prayers	19 (14.7)	41 (18.6)	60 (17.1)	
Spirituality	4 (3.1)	12 (5.4)	16 (4.6)	
Other	4 (3.1)	7 (3.2)	11 (3.1)	
Don’t know	18 (14.0)	21 (9.5)	39 (11.1)	

*Multiple responses allowed

** P-values were derived using Pearson’s chi-square test for categorical variables. Fisher’s exact test was applied where expected cell counts were <5.

### Barriers to treatment and perceptions of epilepsy

The main obstacle to treatment was a lack of awareness of the diagnosis (37.8%), especially in Shai-Osudoku (49.5%). Other obstacles included medication costs, doubts about effectiveness, stigma, limited healthcare access, and a preference for alternative medicine.

Significant knowledge gaps persist in the understanding of epilepsy. A higher proportion of participants (39.7%) o were unsure of what caused epilepsy. A substantial portion (36.8%) attributed epilepsy to witchcraft or supernatural forces, while a smaller group (18.5%) correctly identified it as a medical condition.

Among confirmed epilepsy cases, significantly more participants in Ningo-Prampram had ever consulted a priest/pastor/man of God for prayers or deliverance than in Shai-Osudoku (82.6% vs 70.5%; p = 0.005). Similarly, beliefs about whether epilepsy could be cured also differed significantly between districts (p = 0.016), with a higher proportion in Shai-Osudoku reporting that it could be cured (87.0% vs 77.2%). However, no significant district-level differences were observed in perceived causes of epilepsy (*p* = 0.082) or preferred treatment options among participants who believed epilepsy could be cured (*p* = 0.632). Most (83%) believed the condition was treatable. Of these, 63% viewed ASM as the most effective treatment, while 17% considered prayer as most effective. The treatment history and associated factors are presented in Table 2.

## Discussion

We examined epilepsy management within the Shai-Osudoku and Ningo-Prampram districts in Ghana. The treatment gap, defined as the proportion of people with epilepsy who were not on ASM during the reference period, was high and broadly consistent with estimates from other SSA settings [15,26,27]. However, comparisons should be interpreted cautiously because definitions of the treatment gap vary across studies, with some incorporating treatment adequacy, continuity of care, or effective biomedical management rather than recent ASM use alone. Consequently, the gap we report captures recent access to medication. This may overestimate the proportion of individuals receiving adequate care when compared to studies that define the gap based on seizure control or treatment appropriateness. We also observed geographic disparities in untreated epilepsy, with a marked gap in the predominantly rural Shai-Osudoku district. This difference is consistent with reports of higher treatment gaps in rural areas [8].

Several factors were associated with the observed treatment gap, and these were multifaceted. Lack of awareness was frequently reported and was associated with the diagnostic gap observed in this study, consistent with findings among people with epilepsy in a suburban area of Southeast Nigeria [27]. These observations align with evidence suggesting that low awareness is associated with reduced access to care in many rural African communities [28,29].

The substantial treatment gap we observed, which reflects recent ASM use, is consistent with ongoing challenges in epilepsy care delivery reported in Ghana and across SSA [5,16,17]. In areas like Shai-Osudoku, this gap persists despite an existing network of health facilities and may be associated with multiple barriers along the treatment pathway. These include limited knowledge among primary healthcare workers, delayed early treatment-seeking, insufficient numbers of specialists, weak diagnostic capacity and inadequate diagnostic tools. These factors may be associated with the undermanagement of epilepsy in these settings. Reducing the treatment gap in Ghana and similar SSA contexts may require urgent, coordinated action across the public health, community, and policy sectors to expand treatment coverage. In particular, targeted training programmes for primary healthcare workers could play an important role in improving diagnostic accuracy and enabling the initiation of treatment [16].

Beyond individual and cultural barriers, health system weaknesses were also associated with the treatment gap. Primary healthcare facilities in the study area often lack personnel trained in epilepsy diagnosis and management; essential ASMs are available inconsistently, and referral pathways are underdeveloped. Supply-chain disruptions and limited procurement budgets may be associated with drug stock-outs, particularly in the more rural Shai-Osudoku district. Task-sharing models, in which non-specialist health workers are trained to diagnose and manage epilepsy under supervision, have shown promise in comparable low-resource settings and may be associated with reduced workforce constraints [30]. Strengthening primary care capacity, improving ASM procurement and distribution, and integrating epilepsy care into existing health programmes have been proposed as strategies to address these structural barriers [31].

Consistent with many African reports, our findings suggest that deeply ingrained cultural beliefs may be associated with epilepsy treatment-seeking behaviours [14,16,17]. Attributing epilepsy to spiritual causes, such as witchcraft or curses, was associated with a greater reliance on traditional medicine and spiritualists. This perspective often co-exists with biomedical care, which may be viewed as complementary rather than primary [13]. Such pluralistic health-seeking was associated with delays in seeking biomedical treatment, interruptions in care or nonadherence to prescribed medications. Reliance on traditional methods may also be associated with epilepsy-related stigma, which, in turn, may be associated with healthcare-seeking behaviour [15]. These underscore the need for public health interventions that integrate culturally sensitive, community-based education to improve epilepsy awareness, reduce stigma, and engage with existing belief systems [17]. Rather than dismissing traditional community structures, interventions could foster collaborative pathways that respect pluralistic care models and may help facilitate effective care.

Dependence on alternative care was also associated with financial barriers, with almost a quarter of participants citing high ASM costs, followed by pharmacy stock-outs, particularly in remote areas. This situation was further associated with reports that some traditional practitioners actively discredit the effectiveness of biomedical treatment. These findings are consistent with evidence from Kenya that high medication costs and limited ASM availability are important barriers to treatment access [26,32]. Such barriers may be associated with nonadherence or even treatment discontinuation [16,17]. In resource-limited settings, such as Ghana and other SSA contexts, strengthening health systems and expanding affordable, consistent ASM access, particularly in rural areas, may be associated with improved care continuity.

The study has several limitations. First, households that were unavailable for screening were excluded after three contact attempts, but this may still have introduced attrition bias. Second, the multi-stage screening design resulted in decreasing numbers at each stage. Not all reductions across screening stages represented attrition, as some participants screened negative at Stage 2 and were appropriately excluded from further neurological assessment. However, loss to follow-up among eligible participants may have introduced some uncertainty in prevalence and treatment gap estimates. Despite the application of inverse probability weighting, residual attrition bias may remain if individuals lost to follow-up differed systematically from completers in terms of healthcare access, stigma, or seizure severity. Such differences could influence estimates of prevalence and the treatment gap.

Thirdly, diagnostic misclassification is possible. The screening tool, although locally validated [23], may have limited sensitivity for non-convulsive epilepsies, and some seizure mimics may have been misclassified as epilepsy. However, efforts were made to mitigate this with physician confirmation of cases. Fourth, several key variables-including ASM adherence, seizure frequency, and treatment effectiveness were based on self-report. This introduces potential recall bias and social desirability bias. Objective measures such as serum drug levels were not obtained due to logistical constraints. Fifth, the cross-sectional design precludes causal inference. Lastly, resource constraints limited the capacity to conduct independent validation of prevalence and treatment gap estimates, and findings may not be fully generalizable beyond the districts.

## Conclusion

We identified a substantial epilepsy treatment gap in Ghana, underlined by a confluence of awareness gaps, sociocultural, and economic hurdles. Improving this requires a multidisciplinary approach beyond a purely medical model. Sustainable management, aligned with global frameworks such as the WHO Mental Health Gap Action Programme (mhGAP) and the Intersectoral Global Action Plan on Epilepsy (IGAP), can improve outcomes. Key strategies include community education, primary healthcare worker training, policy reforms and collaborative engagement with traditional healers. Integrating epilepsy care into primary health systems through task-sharing, as recommended by IGAP, is critical. Lastly, robust ASM supply-chain mechanisms must be implemented.

## Supporting information

S1 File EPInA Ghana survey households. (CSV)

S2 File EPInA Ghana survey members. (CSV)
