# Supplementary material for: Barriers to epilepsy care: Measuring the treatment gap in rural and peri-urban districts in Ghana
Source: PLoS One. 2026 Aug 11;21(8):e0355854. doi: 10.1371/journal.pone.0355854 (PMC13460580; doi:10.1371/journal.pone.0355854)
Supplement: Supplementary file 1 [file pone.0355854.s004.pdf]

PONE-D-26-03687
Barriers to Epilepsy Care: Measuring the Treatment Gap in Rural and Peri-Urban Districts in Ghana.

Response to editorial comments

We thank the Editor for these important observations and have revised the manuscript accordingly.

Comment 1: Please ensure that your manuscript meets PLOS ONE's style requirements, including those for file naming.

Response: *Thank you. We have revised the manuscript to conform to PLOS ONE formatting requirements, including file naming, manuscript structure, figure presentation, and title page formatting.*

Comment 2-5: Journal Requirement - Funding statement, NIHR expansion, and role of funders

Response: *Thank you for this observation. We have removed funding-related text from the Acknowledgments section and from the manuscript and updated the Funding Statement accordingly. We have also expanded the acronym "NIHR" as National Institute for Health and Care Research.*

We have revised the funding statement: *"The UK National Institute for Health and Care Research (NIHR 200134) funded this project through Official Development Assistance (ODA). The views expressed in this publication are those of the authors and not necessarily those of the National Health Service (NHS), the NIHR, or the Department of Health and Social Care*".

Comment 6: Data availability

Response: *Thank you. We confirm that the minimal anonymized dataset underlying the findings was prepared and uploaded as Supporting Information files in the initial submission.*

Comment 7: Your ethics statement should only appear in the Methods section of your manuscript.

Response: *Thank you for this observation. We have revised the manuscript accordingly by moving the ethics statement section to the Methods section of the manuscript and removing it from other*

sections to ensure consistency with the journal requirements. The necessary changes have been made in the manuscript (Page 12, lines 239-248).

Comment 8: Please ensure that you refer to Figure 1 in your text as, if accepted, production will need this reference to link the reader to the figure.

Response: *Thank you for this observation. We have now appropriately cited Figure 1 in the main text of the manuscript to ensure proper linkage between the text and the figure during production.*

Comment 9: We note that Figure 1 in your submission contains map images which may be copyrighted.

Response: *Thank you for this important observation. The maps presented in Figure 1 were generated by the authors using geographic coordinates collected from the study area and administrative shapefiles obtained from the respective district assemblies (local authority). No copyrighted Google Maps, Google Earth, Street View images, or other proprietary satellite imagery were used in the preparation of the figure.* This figure has been published by the same authors elsewhere (1). *This has now been properly referenced (Page 7, line 132).*

1. Darkwa EK, Asiamah S, Awini E, Sottie C, Godi A, Williams JE, et al. The prevalence and determinants of epilepsy in Ghana: A population-based study in two districts using a three-stage approach. Epilepsia. 2026.

Response to Reviewers

We thank the reviewers for their useful comments, and we have revised the manuscript as such

Reviewer # 1

Comment: The authors failed to state in the Methods section that the study was granted ethics committee approval, although the approval letters were shown under "Supporting Information".

Response: *Thank you for this comment. The ethics approval statement was included in the original manuscript; however, it was inadvertently placed before the References section. We have now revised the manuscript by moving the ethics statement to the Methods section, where it is more appropriately positioned in line with the journal requirements. The necessary changes have been made in the manuscript (Page 12, lines 239-248).*

Comment: Although the statistical analysis was performed appropriately and rigorously, the authors failed to state in the text under "Results" which statistical method they used (eg, Fisher's exact test, chi-square test). They also failed to state some important results in the text and only displayed them on two tables.

Response: *Thank you. We have revised the manuscript accordingly by explicitly stating the statistical tests used in the analyses within the Methods section, including the appropriate tests applied for comparisons between groups (Page 11, lines 224-226).*

In addition, key findings previously presented only in the tables have now been incorporated into the narrative text of the Results section to improve clarity and interpretation. The necessary changes have been made in the manuscript (Pages 12-13 and 14, lines 256-258, 261-266, 279-281, and lines 298-305).

Reviewer # 2

Comments 1: Treatment gap definition and operationalization

The manuscript defines the epilepsy treatment gap appropriately in conceptual terms; however, its operational definition within the study requires clarification. In practice, treatment status appears to rely largely on reported ASM use in the preceding month, which may not adequately distinguish between untreated epilepsy, undertreatment, interrupted treatment, or poor adherence. Greater clarity regarding how 'appropriate treatment' was assessed would strengthen the validity and interpretability of the treatment-gap estimates.

Response: *Thank you for this important observation. We have revised the manuscript to provide greater clarity on the operational definition of the epilepsy treatment gap used in this study. Specifically, we have expanded the Methods section to explain how treatment status and 'appropriate treatment' were assessed, including the role of recent anti-seizure medication (ASM) use, treatment continuity, and clinical history obtained during the assessment. We have also clarified the limitations associated with relying on self-reported ASM use in the preceding month, particularly regarding the distinction between untreated epilepsy, undertreatment, interrupted treatment, and poor adherence (Pages 10, lines 194-199, Page 20, lines 392-397).*

Comment 2: Cross-sectional design and causal interpretation

Several sections of the discussion imply causal relationships between sociocultural beliefs, awareness deficits, and treatment gaps. However, given the cross-sectional observational design, the findings should be interpreted as associations rather than evidence of causality. The discussion would benefit from greater caution, using terms such as 'associated with' or 'may contribute to' rather than implying direct causal effects.

Response: *Thank you. We agree that, given the cross-sectional observational design of this study, causal inferences cannot be established. The Discussion section has been carefully revised to avoid causal language and to ensure that findings are interpreted as associations rather than evidence of direct causality. Terms implying causal relationships, such as "led to," "resulted in," or "caused," have been replaced with more appropriate cautious language such as "was associated with," "may contribute to," or "could partly explain" (Pages 17, 18 and 19, lines 322-334 and 350-372).*

Comment 3: Diagnostic ascertainment and epilepsy misclassification

The multistage screening design with physician confirmation is a major strength; however, additional details regarding validation of the screening tool would improve methodological rigor. Information on sensitivity, specificity, and diagnostic reliability is lacking. Furthermore, community-based surveys in low-resource settings may underdetect nonconvulsive epilepsies and may overdetect seizure mimics such as syncope or psychogenic nonepileptic events.

Response: *Thank you for acknowledging the strength of the multistage screening design. We appreciate this important comment. In response, we have included additional details in the manuscript regarding the validation of the screening tool and provided an appropriate reference to the validation study conducted before this household survey to strengthen the methodological rigor of the paper (Page 8, lines 151-155).*

We also acknowledge the potential limitations of community-based epilepsy surveys in low-resource settings, including the possible under detection of nonconvulsive epilepsies and the over-detection of seizure mimics such as syncope or psychogenic nonepileptic events. To minimize these risks, we implemented a multistage screening process, employed trained fieldworkers for

standardized data collection, and ensured neurologist confirmation of suspected epilepsy cases at the final stage of assessment. These measures were intended to improve diagnostic accuracy and reduce the likelihood of misclassification. Relevant clarifications have now been incorporated into the Methods and Discussion sections of the revised manuscript (Pages 7 and 20, lines 160-163 and 388-391).

Comment 4: Attrition bias and representativeness

A substantial proportion of suspected cases did not complete all stages of assessment, raising the possibility of attrition bias despite the use of inverse probability weighting. Individuals lost during follow-up may systematically differ in healthcare access, stigma, or seizure severity, potentially influencing both prevalence and treatment-gap estimates. This limitation should be discussed in greater depth.

Response: *Thank you. We acknowledge that loss to follow-up across screening stages could have introduced bias and led to underestimation of epilepsy prevalence and treatment gap estimates. To address this, we applied attrition-adjusted prevalence estimation using inverse probability weighting (IPW) to account for participants lost at Stages 2 and 3. Sampling weights were derived from the inverse probability of progression through each screening stage, based on observed characteristics and response patterns. These weights were incorporated into prevalence calculations to reduce attrition bias, improve representativeness of confirmed epilepsy cases, and strengthen the robustness of the estimates. This limitation has now been discussed in greater detail (Pages 20, lines 380-387).*

Comment 5: Reliance on self-reported data

Several important variables, including medication adherence, seizure control, treatment effectiveness, and health-seeking behavior, relied primarily on participant self-report. This introduces the possibility of recall bias and social desirability bias, particularly in relation to adherence and perceived seizure outcomes. The limitations associated with self-reported treatment data should therefore be emphasized more explicitly.

Response: *Thank you. We agree that reliance on self-reported data for key variables such as medication adherence, seizure control, treatment effectiveness, and health-seeking behaviour may introduce recall bias and social desirability bias. In response, we have strengthened the Discussion/Limitations section to more explicitly acknowledge these potential sources of bias and their possible influence on the interpretation of treatment-related findings. We now clarify that treatment-related information was primarily based on participant or caregiver self-report, which may have affected the accuracy of reported adherence, seizure outcomes, and care-seeking patterns. These limitations have now been explicitly discussed in the revised manuscript (Page 20, lines 392-397).*

Comment 6: Interpretation of sociocultural beliefs

The discussion appropriately highlights the importance of sociocultural beliefs in epilepsy care pathways; however, some interpretations risk oversimplifying culturally embedded explanatory models as mere misinformation. Greater engagement with literature on pluralistic healthcare systems and culturally informed health-seeking behaviors would strengthen the discussion and provide a more balanced interpretation of traditional and spiritual beliefs.

Response: *Thank you. We appreciate the reviewer's emphasis on the need for a more balanced interpretation of sociocultural beliefs within epilepsy care pathways. We agree that traditional and spiritual beliefs should not be framed solely as misinformation, as health-seeking behaviours in many low-resource settings often occur within pluralistic healthcare systems where biomedical, traditional, and spiritual approaches may coexist and shape care pathways. In response, we have revised the Discussion section to adopt a more nuanced interpretation of culturally embedded explanatory models and strengthened the discussion by incorporating relevant literature on pluralistic healthcare systems and culturally informed health-seeking behaviours. (Pages 18, lines 350-363).*

Comment 7: Limited health-system analysis

Although the manuscript identifies important barriers such as medication cost, drug shortages and limited specialist access, the health-system analysis remains relatively underdeveloped.

Additional discussion of primary healthcare capacity, ASM supply-chain challenges, workforce limitations, and economic barriers would strengthen the policy relevance of the findings.

Response: *Thank you. We agree that expanding the health-system perspective would strengthen the policy relevance of the findings. In response, we have revised the Discussion section to provide a more comprehensive interpretation of the structural and health-system barriers that may influence epilepsy diagnosis, treatment access, and continuity of care. Specifically, we have expanded the discussion to include primary healthcare capacity for epilepsy management, anti-seizure medication (ASM) supply-chain and availability challenges, workforce limitations including restricted access to specialist neurological care. (Page 18, lines 340-349).*

Comment 8: Statistical analysis and reporting

The statistical methods are generally appropriate; however, additional detail regarding handling of clustering, missing data, and potential confounders would improve transparency.

Response: *Thank you. To account for potential household and community-level clustering inherent in the door-to-door sampling design, cluster-robust standard errors were calculated for all comparative analyses. Potential confounding by age and sex was controlled through exact matching during the case-control selection phase (Page 10 and 11, lines 228-235).*

Comment 9: References

The reference list is generally appropriate and includes several authoritative sources on epilepsy epidemiology, treatment gaps, and sub-Saharan African epilepsy care. The strong use of regional African literature is a notable strength and provides relevant contextual grounding for the findings. However, the manuscript would benefit from inclusion of more recent global policy and implementation-science references, particularly WHO mhGAP and IGAP frameworks, task-sharing models, and literature addressing explanatory models of health seeking in epilepsy.

Response: *Thank you for the suggestions. We have revised the manuscript to include more recent global policy and implementation-science references, particularly the WHO Mental Health Gap Action Programme (mhGAP), the Intersectoral Global Action Plan on Epilepsy and Other Neurological Disorders (IGAP 2022–2031), task-sharing approaches, and literature on*

explanatory models that influences health-seeking behaviour in epilepsy.(Page 18. Lines 344-349).

Comment 10: Recommendations

The recommendations would benefit from stronger linkage to existing implementation frameworks such as WHO mhGAP and IGAP initiatives, as well as evidence from task-sharing epilepsy-care models in similar low-resource settings.

Response: *Thank you for this valuable comment. We have strengthened the recommendations section by explicitly linking our findings to existing implementation frameworks, including the WHO Mental Health Gap Action Programme (mhGAP) and the Intersectoral Global Action Plan on Epilepsy and Other Neurological Disorders (IGAP 2022–2031). (Page 21, lines 408-417).*

Supplementary Comments (word document)

Comment 1:

1. Under "Methods – Study setting", lines 114-118 (Page 5):

   The authors listed 16 health facilities in Shai-Osudoku District, but only stated "fewer health facilities" for Ningo-Prampram District. There is a need to mention the health facilities in Ningo-Prampram for consistency.

   Response: *Thank you for the suggestion. The number of facilities in Ningo-Prampram District have now been updated in the manuscript for consistency (Page 6, lines 123-125).*

Comment 2:

2. Under "Methods – Study design", line 132 (Page 6):

   The authors stated "the project began in 2019 and ended in January 2025". Authors should state the exact date and month for both years.

   Response: *Thank you for the comment. We have revised the manuscript to provide the exact start and end dates of the project for clarity and consistency. The project commenced on October 1, 2019, and was completed on January 31, 2025 (Pages 7, lines 142-143).*

Comment 3:

3. Although ethics committee approvals for the study are shown under "Supporting Information", the authors failed to mention in the "Methods" section that they were granted ethics committee approval before commencing the study. Authors should mention it and quote the reference numbers and dates of approvals/renewals.

Response: Thank you. *Ethical approval was obtained before study commencement in Ghana from the Ghana Health Service Ethics Review Committee (GHS-ERC 022/08/20; approved 14 December 2020) and the Dodowa Health Research Centre Institutional Review Board (DHRCIRB 95/08/20; approved 20 November 2020). Annual renewals were obtained throughout the study period (GHS-ERC: 14 December 2021, 2022, and 2023; DHRCIRB: 15 November 2021, 8 December 2022, and 22 January 2024) (Page 12, lines 239-248).*

Comment 5:

4. There was no mention of sample size calculation in the "Methods" section. While 55,000 participants exceed the minimum sample size required for most studies, there is still a need for context on the total population compared to number of participants. So, at least, authors should state the most recent combined population of the two study areas.

Response: *Thank you. We used a census approach within the Dodowa Health and Demographic Surveillance System (DHDSS), covering eligible 136,727 residents in the Shai-Osudoku and Ningo-Prampram surveillance areas. The combined DHDSS population served as the sampling frame, with 65,177 participants screened during the study period. All confirmed epilepsy cases identified during the survey were included in the study, resulting in a final sample of 421 participants (Page 9, lines 172-175).*

Comment 6:

5. Under “Methods – Statistical analysis”, line 193 (Page 9):

   Authors opened a square bracket enclosing [Stata/SE17. . . . ., but didn’t close it. Authors should close the square bracket or delete it and move back the parenthesis beginning at “StataCorp” to also enclose “Stata/SE17”.

   Response: *We thank the reviewer for pointing out this formatting issue. As suggested, we have moved the parentheses back so that they now enclose “Stata/SE17” together with “StataCorp.” (Page 11, line 222).*

   Comment 7:

6. Under “Results – Prevalence of epilepsy”, lines 201-206 (Page 9):

   The initial screening identified 617 suspected cases of epilepsy, of which 544 were evaluated at Stage 2, and 73 were left out. The third stage (clinical assessment) also left out 32 of 467 participants with suspected epilepsy. Thus, a total 105 (17.01%) of 617 suspected epilepsy cases missed evaluation at Stage 2 or Stage 3. Failing to evaluate 17% participants with suspected epilepsy could cause significant under-estimates of epilepsy prevalence and treatment gap reported in the study.

   Although the authors may have mitigated that with “attrition-adjusted prevalence” mentioned in line 206, they should explain the steps and the scientific basis.

   Authors should also explain the high attrition rate. If all participants had given informed consent at survey interviews, why did 17% of those with suspected epilepsy missed further evaluation and clinical assessment by neurologists? Did stigma and misconceptions prevent them attending evaluation at the health clinic? Although this was discussed broadly under “Discussion” section, authors didn’t specifically address the 17% drop-out rate.

   Response: Thank *you. First, we would like to clarify that not all participants who did not proceed to Stage 3 were lost to follow-up. The reduction in participant numbers between stages reflected both true attrition (non-completion of follow-up assessments) and expected exclusions based on the study’s multi-stage screening design. Specifically, some participants who screened positive at Stage 1 subsequently screened negative at Stage 2 and were therefore*

not eligible for Stage 3 neurological assessment. At stage 1, the questionnaire was administered to household heads, adult mothers, or knowledgeable household members, for all household usual members including those temporarily absent at the times of the visit. At Stage 2, individuals screening positive at Stage 1 received a follow- up by field supervisors at home to enhance specificity.

Importantly, not all reductions between screening stages reflected loss to follow-up. Of the 544 participants assessed at Stage 2, 72 (13.2%) screened negative and were excluded from Stage 3 clinical assessment as false positives from the initial broad screening tool, consistent with the multi-stage study design. Therefore, the observed reduction in participant numbers should not be interpreted solely as attrition. The flow of participants during recruitment has been reported elsewhere (1)

This has been clarified in the methods (Page 7, lines 162-163 and Page 9, lines 172-175), Results (Page 12, lines 263-266), and Discussions (Page 20, lines 380-387).

1. Darkwa EK, Asiamah S, Awini E, Sottie C, Godi A, Williams JE, et al. The prevalence and determinants of epilepsy in Ghana: A population-based study in two districts using a three-stage approach. Epilepsia. 2026.

Comment 8:

7. Lines 232-233 (Page 10-11):

"The majority (39.7%) of the cases were unsure of their cause" is ambiguous and false, as 39.7% is not a majority. "Their cause" also contradicts the singular noun, "epilepsy".

Authors may rephrase the sentence as: "A high proportion of participants (39.7%) were unsure of what caused epilepsy".

Response: *Thank you for this observation. We agree that the term "majority" was inaccurate, as 39.7% does not constitute a majority, and that the phrase "their cause" was unclear and grammatically inconsistent with the singular noun epilepsy. The sentence has been revised for accuracy, clarity, and grammatical consistency. (Page 14, lines 294-295).*

Comment 9:

8. Results, Table 1, Table 2, lines 201-239 (Pages 9-12):

   Although authors showed P values on Table 1 and Table 2, they didn't state the statistical methods of comparison in the text or on the tables. If they used a chi-square test or Fisher's exact test, this should be stated in the text under "Result" section, where appropriate (with the relevant percentages, chi-square statistic and the P value all quoted within parentheses according to standard protocol).

   Authors are advised to look for examples in other publications on how to properly report chi-square tests of association under "Results".

Response: *Thank you for this important observation. We agree that the statistical methods used to generate the p-values presented in Tables 1 and 2 were not explicitly stated in the original manuscript. We have now revised the manuscript to improve clarity and align with standard reporting practices.*

Specifically, we have clarified in the Methods section that comparisons between districts for categorical variables were performed using Pearson's chi-square test, while Fisher's exact test was applied where expected cell counts were small (<5). In addition, footnotes have now been added to Tables 1 and 2 to indicate the statistical tests used to derive the reported p-values.

Furthermore, key statistically significant comparisons have been made in the Results section, with the relevant percentages, test statistics, and p-values reported where appropriate. Methods (Page 11, lines 224-226), Results (Page 13,14 and 15, lines 256-266, 279-288 and 298-304).

Prof A Akpalu

On behalf of the authors
