# Supplementary material for: Barriers to epilepsy care: Measuring the treatment gap in rural and peri-urban districts in Ghana
Source: PLoS One. 2026 Aug 11;21(8):e0355854. doi: 10.1371/journal.pone.0355854 (PMC13460580; doi:10.1371/journal.pone.0355854)
Supplement: Supplementary file 2 [file pone.0355854.s005.pdf]

Manuscript ID: PONE-D-26-03687R1 : *Barriers to Epilepsy Care: Measuring the Treatment Gap in Rural and Peri-Urban Districts in Ghana*

Response to Reviewers

Dear Hiroki Annaka,

We thank the Academic Editor and reviewers for their evaluation of our revised manuscript and for their feedback. We appreciate that the reviewers found the manuscript to have improved. We have carefully considered the remaining comments and revised the manuscript accordingly. Our reactions are provided below. We have used the opportunity to address minor issues identified in the manuscript in track changes.

Reviewer #1

We thank the reviewer for the positive assessment of our manuscript and appreciate the time and effort invested in reviewing our work.

Reviewer #2

We thank the reviewer for the positive evaluation of our revised manuscript and for the remaining suggestions. We have now addressed each comment.

Comment 1:Treatment gap definition: Although the operational definition has been clarified, the reported treatment gap reflects recent ASM use rather than treatment adequacy. This distinction should remain explicit when interpreting the findings and comparing them with other studies.

Response: *Thank you for this observation. We agree that our estimate reflects* ***recent antiseizure medication (ASM) use*** *and should not be interpreted as a measure of treatment adequacy. We have revised the Discussion to explicitly state that the treatment gap reported in this study is based on recent ASM use reflecting lack of current treatment rather than the overall adequacy or quality of epilepsy management. We have also emphasized this distinction when comparing our findings with previous studies that may have used different operational definitions of the treatment gap.* (Page 16, lines 301-310)

Comment 2: Interpretation of findings: The discussion should continue to avoid causal language. Given the cross-sectional design, factors such as lack of awareness, sociocultural beliefs, and reliance on traditional medicine should be described as being associated with, rather than causing, the treatment gap.

Response: *We appreciate this comment. We have carefully reviewed the Discussion and replaced any remaining causal wording with language appropriate for a cross-sectional study. Throughout the manuscript, statements implying causation have been revised to describe these factors as being* ***associated with*** *the treatment gap rather than causing it. This ensures that the interpretation of our findings accurately reflects the observational nature of the study.* (Page 16-19, line 300-367).

Sincerely,

Prof. Albert Akpalu, on behalf of the author team
