# Supplementary material for: Barriers to epilepsy care: Measuring the treatment gap in rural and peri-urban districts in Ghana
Source: PLoS One. 2026 Aug 11;21(8):e0355854. doi: 10.1371/journal.pone.0355854 (PMC13460580; doi:10.1371/journal.pone.0355854)
Supplement: Supplementary file 3 [file pone.0355854.s003.docx]

**Reviewer Comments**

1. Under “Methods – Study setting”, lines 114-118 (Page 5):

The authors listed 16 health facilities in Shai-Osudoku District, but only stated “fewer health facilities” for Ningo-Prampram District. There is a need to mention the health facilities in Ningo-Prampram for consistency.

2. Under “Methods – Study design”, line 132 (Page 6):

The authors stated “the project began in 2019 and ended in January 2025”. Authors should state the exact date and month for both years.

3. Although ethics committee approvals for the study are shown under “Supporting Information”, the authors failed to mention in the “Methods” section that they were granted ethics committee approval before commencing the study. Authors should mention it and quote the reference numbers and dates of approvals/renewals.
4. There was no mention of sample size calculation in the “Methods” section. While 55,000 participants exceed the minimum sample size required for most studies, there is still a need for context on the total population compared to number of participants. So, at the least, authors should state the most recent combined population of the two study areas.
5. Under “Methods – Statistical analysis”, line 193 (Page 9):

Authors opened a square bracket enclosing [Stata/SE17. . . . ., but didn’t close it. Authors should close the square bracket or delete it and move back the parenthesis beginning at “StataCorp” to also enclose “Stata/SE17”.

6. Under “Results – Prevalence of epilepsy”, lines 201-206 (Page 9):

The initial screening identified 617 suspected cases of epilepsy, of which 544 were evaluated at Stage 2, and 73 were left out. The third stage (clinical assessment) also left out 32 of 467 participants with suspected epilepsy. Thus, a total 105 (17.01%) of 617 suspected epilepsy cases missed evaluation at Stage 2 or Stage 3. Failing to evaluate 17% participants with suspected epilepsy could cause significant under-estimates of epilepsy prevalence and treatment gap reported in the study.

Although the authors may have mitigated that with “attrition-adjusted prevalence” mentioned in line 206, they should explain the steps and the scientific basis.

Authors should also explain the high attrition rate. If all participants had given informed consent at survey interviews, why did 17% of those with suspected epilepsy missed further evaluation and clinical assessment by neurologists? Did stigma and misconceptions prevent them attending evaluation at the health clinic? Although this was discussed broadly under “Discussion” section, authors didn’t specifically address the 17% drop-out rate.

7. Lines 232-233 (Page 10-11):

“The majority (39.7%) of the cases were unsure of their cause” is ambiguous and false, as 39.7% is not a majority. “Their cause” also contradicts the singular noun, “epilepsy”.

Authors may rephrase the sentence as: “A high proportion of participants (39.7%) were unsure of what caused epilepsy”.

8. Results, Table 1, Table 2, lines 201-239 (Pages 9-12):

Although authors showed P values on Table 1 and Table 2, they didn’t state the statistical methods of comparison in the text or on the tables. If they used a chi-square test or Fisher’s exact test, this should be stated in the text under “Result” section, where appropriate (with the relevant percentages, chi-square statistic and the P value all quoted within parentheses according to standard protocol).

Authors are advised to look for examples in other publications on how to properly report chi-square tests of association under “Results”.
