# Supplementary material for: Barriers to epilepsy care: Measuring the treatment gap in rural and peri-urban districts in Ghana
Source: PLoS One. 2026 Aug 11;21(8):e0355854. doi: 10.1371/journal.pone.0355854 (PMC13460580; doi:10.1371/journal.pone.0355854)
Supplement: Peer review history

# Peer review: Decision Letter 0

**DOI:** 10.1371/journal.pone.0355854.r001

**Authors:** Hiroki Annaka

10 May 2026

Dear Dr. Akpalu,

Thank you for submitting your manuscript to PLOS ONE. After careful consideration, we feel that it has merit but does not fully meet PLOS ONE’s publication criteria as it currently stands. Therefore, we invite you to submit a revised version of the manuscript that addresses the points raised during the review process.

==============================

==============================

Please submit your revised manuscript by Jun 24 2026 11:59PM. If you will need more time than this to complete your revisions, please reply to this message or contact the journal office at plosone@plos.org. When you're ready to submit your revision, log on to https://www.editorialmanager.com/pone/ and select the 'Submissions Needing Revision' folder to locate your manuscript file.

- A letter that responds to each point raised by the academic editor and reviewer(s). You should upload this letter as a separate file labeled 'Response to Reviewers'.
- A marked-up copy of your manuscript that highlights changes made to the original version. You should upload this as a separate file labeled 'Revised Manuscript with Track Changes'.
- An unmarked version of your revised paper without tracked changes. You should upload this as a separate file labeled 'Manuscript'.

If you would like to make changes to your financial disclosure, please include your updated statement in your cover letter. Guidelines for resubmitting your figure files are available below the reviewer comments at the end of this letter.

If applicable, we recommend that you deposit your laboratory protocols in protocols.io to enhance the reproducibility of your results. Protocols.io assigns your protocol its own identifier (DOI) so that it can be cited independently in the future. For instructions see: https://journals.plos.org/plosone/s/submission-guidelines#loc-laboratory-protocols. Additionally, PLOS ONE offers an option for publishing peer-reviewed Lab Protocol articles, which describe protocols hosted on protocols.io. Read more information on sharing protocols at https://plos.org/protocols?utm_medium=editorial-email&utm_source=authorletters&utm_campaign=protocols.

As the corresponding author, your ORCID iD is verified in the submission system and will appear in the published article. PLOS supports the use of ORCID, and we encourage all coauthors to register for an ORCID iD and use it as well. Please encourage your coauthors to verify their ORCID iD within the submission system before final acceptance, as unverified ORCID iDs will not appear in the published article. *Only*  the individual author can complete the verification step; PLOS staff *cannot*  verify ORCID iDs on behalf of authors.

We look forward to receiving your revised manuscript.

Kind regards,

Hiroki Annaka

Academic Editor

PLOS One

Journal Requirements:

When submitting your revision, we need you to address these additional requirements.

1. Please ensure that your manuscript meets PLOS ONE's style requirements, including those for file naming. The PLOS ONE style templates can be found at

https://journals.plos.org/plosone/s/file?id=wjVg/PLOSOne_formatting_sample_main_body.pdf and

https://journals.plos.org/plosone/s/file?id=ba62/PLOSOne_formatting_sample_title_authors_affiliations.pdf

2. Thank you for stating the following in the Acknowledgments Section of your manuscript:

“This project was commissioned by the National Institute for Health Research (NIHR 200134) under Official Development Assistance (ODA) funding. This grant supports EKD. The views expressed in this publication are those of the author(s) and not necessarily those of the NHS, the National Institute for Health Research or the Department of Health and Social Care.”

We note that you have provided funding information that is not currently declared in your Funding Statement. However, funding information should not appear in the Acknowledgments section or other areas of your manuscript. We will only publish funding information present in the Funding Statement section of the online submission form.

Please remove any funding-related text from the manuscript and let us know how you would like to update your Funding Statement. Currently, your Funding Statement reads as follows:

“NIHR grant 200134”

Please include your amended statements within your cover letter; we will change the online submission form on your behalf.

3. We note that the grant information you provided in the ‘Funding Information’ and ‘Financial Disclosure’ sections do not match.

When you resubmit, please ensure that you provide the correct grant numbers for the awards you received for your study in the ‘Funding Information’ section.

4. Thank you for stating the following financial disclosure:

“NIHR grant 200134”

Please state what role the funders took in the study.  If the funders had no role, please state: "The funders had no role in study design, data collection and analysis, decision to publish, or preparation of the manuscript."

If this statement is not correct you must amend it as needed.

Please include this amended Role of Funder statement in your cover letter; we will change the online submission form on your behalf.

5. Please expand the acronym “NIHR” (as indicated in your financial disclosure) so that it states the name of your funders in full.

This information should be included in your cover letter; we will change the online submission form on your behalf.

6. We note that your Data Availability Statement is currently as follows: “All relevant data are within the manuscript and its Supporting Information files.”

Please confirm at this time whether or not your submission contains all raw data required to replicate the results of your study. Authors must share the “minimal data set” for their submission. PLOS defines the minimal data set to consist of the data required to replicate all study findings reported in the article, as well as related metadata and methods (https://journals.plos.org/plosone/s/data-availability#loc-minimal-data-set-definition).

For example, authors should submit the following data:

- The values behind the means, standard deviations and other measures reported;

- The values used to build graphs;

- The points extracted from images for analysis.

Authors do not need to submit their entire data set if only a portion of the data was used in the reported study.

If your submission does not contain these data, please either upload them as Supporting Information files or deposit them to a stable, public repository and provide us with the relevant URLs, DOIs, or accession numbers. For a list of recommended repositories, please see https://journals.plos.org/plosone/s/recommended-repositories.

If there are ethical or legal restrictions on sharing a de-identified data set, please explain them in detail (e.g., data contain potentially sensitive information, data are owned by a third-party organization, etc.) and who has imposed them (e.g., an ethics committee). Please also provide contact information for a data access committee, ethics committee, or other institutional body to which data requests may be sent. If data are owned by a third party, please indicate how others may request data access.

7. Your ethics statement should only appear in the Methods section of your manuscript. If your ethics statement is written in any section besides the Methods, please move it to the Methods section and delete it from any other section. Please ensure that your ethics statement is included in your manuscript, as the ethics statement entered into the online submission form will not be published alongside your manuscript.

8. Please ensure that you refer to Figure 1 in your text as, if accepted, production will need this reference to link the reader to the figure.

9. We note that Figure 1 in your submission contain map images which may be copyrighted. All PLOS content is published under the Creative Commons Attribution License (CC BY 4.0), which means that the manuscript, images, and Supporting Information files will be freely available online, and any third party is permitted to access, download, copy, distribute, and use these materials in any way, even commercially, with proper attribution. For these reasons, we cannot publish previously copyrighted maps or satellite images created using proprietary data, such as Google software (Google Maps, Street View, and Earth). For more information, see our copyright guidelines: http://journals.plos.org/plosone/s/licenses-and-copyright.

We require you to either (1) present written permission from the copyright holder to publish these figures specifically under the CC BY 4.0 license, or (2) remove the figures from your submission:

1. You may seek permission from the original copyright holder of Figure(s) [#] to publish the content specifically under the CC BY 4.0 license.

We recommend that you contact the original copyright holder with the Content Permission Form (http://journals.plos.org/plosone/s/file?id=7c09/content-permission-form.pdf) and the following text:

“I request permission for the open-access journal PLOS ONE to publish XXX under the Creative Commons Attribution License (CCAL) CC BY 4.0 (http://creativecommons.org/licenses/by/4.0/). Please be aware that this license allows unrestricted use and distribution, even commercially, by third parties. Please reply and provide explicit written permission to publish XXX under a CC BY license and complete the attached form.”

Please upload the completed Content Permission Form or other proof of granted permissions as an "Other" file with your submission.

In the figure caption of the copyrighted figure, please include the following text: “Reprinted from [ref] under a CC BY license, with permission from [name of publisher], original copyright [original copyright year].”

2. If you are unable to obtain permission from the original copyright holder to publish these figures under the CC BY 4.0 license or if the copyright holder’s requirements are incompatible with the CC BY 4.0 license, please either i) remove the figure or ii) supply a replacement figure that complies with the CC BY 4.0 license. Please check copyright information on all replacement figures and update the figure caption with source information. If applicable, please specify in the figure caption text when a figure is similar but not identical to the original image and is therefore for illustrative purposes only.

The following resources for replacing copyrighted map figures may be helpful:

USGS National Map Viewer (public domain): http://viewer.nationalmap.gov/viewer/

The Gateway to Astronaut Photography of Earth (public domain): http://eol.jsc.nasa.gov/sseop/clickmap/

Maps at the CIA (public domain): https://www.cia.gov/library/publications/the-world-factbook/index.html and https://www.cia.gov/library/publications/cia-maps-publications/index.html

NASA Earth Observatory (public domain): http://earthobservatory.nasa.gov/

Landsat: http://landsat.visibleearth.nasa.gov/

USGS EROS (Earth Resources Observatory and Science (EROS) Center) (public domain): http://eros.usgs.gov/#

Natural Earth (public domain): http://www.naturalearthdata.com/

10. If the reviewer comments include a recommendation to cite specific previously published works, please review and evaluate these publications to determine whether they are relevant and should be cited. There is no requirement to cite these works unless the editor has indicated otherwise.

[Note: HTML markup is below. Please do not edit.]

Reviewers' comments:

Reviewer's Responses to Questions

**Comments to the Author**

1. Is the manuscript technically sound, and do the data support the conclusions?

Reviewer #1: Yes

Reviewer #2: Partly

**********

2. Has the statistical analysis been performed appropriately and rigorously?

Reviewer #1: Yes

Reviewer #2: No

**********

3. Have the authors made all data underlying the findings in their manuscript fully available?

The PLOS Data policy

Reviewer #1: Yes

Reviewer #2: Yes

**********

4. Is the manuscript presented in an intelligible fashion and written in standard English?

Reviewer #1: Yes

Reviewer #2: Yes

**********

Reviewer #1: The authors failed to state in Methods section that the study was granted ethics committee approval, although the approval letters were shown under "Supporting Information".

Although the statistical analysis was performed appropriately and rigorously, the authors failed to state in the text under "Results" which statistical method they used (eg, Fisher's exact test, chi-square test). They also failed to state some important results in the text and only displayed them on two tables.

There are no other issues beside those I mentioned in my comments to authors.

Thank you.

Nura Alkali

Reviewer #2: This is an important and policy-relevant community-based epilepsy epidemiology study from southeastern Ghana addressing treatment gap in sub-Saharan Africa.

The strengths are the large population denominator, staged screening design, focus on treatment barriers, and integration of sociocultural determinants with treatment access data.

The study could be considerably strengthened by addressing the following

1. Treatment gap definition and operationalization

The manuscript defines the epilepsy treatment gap appropriately in conceptual terms; however, its operational definition within the study requires clarification.

In practice, treatment status appears to rely largely on reported ASM use in the preceding month, which may not adequately distinguish between untreated epilepsy, undertreatment, interrupted treatment, or poor adherence. Greater clarity regarding how ‘appropriate treatment’ was assessed would strengthen the validity and interpretability of the treatment-gap estimates.

2. Cross-sectional design and causal interpretation

Several sections of the discussion imply causal relationships between sociocultural beliefs, awareness deficits, and treatment gaps. However, given the cross-sectional observational design, the findings should be interpreted as associations rather than evidence of causality. The discussion would benefit from greater caution, using terms such as ‘associated with’ or ‘may contribute to’ rather than implying direct causal effects.

3. Diagnostic ascertainment and epilepsy misclassification

The multistage screening design with physician confirmation is a major strength; however, additional details regarding validation of the screening tool would improve methodological rigor. Information on sensitivity, specificity, and diagnostic reliability is lacking. Furthermore, community-based surveys in low-resource settings may underdetect nonconvulsive epilepsies and may overdetect seizure mimics such as syncope or psychogenic nonepileptic events.

4. Attrition bias and representativeness

A substantial proportion of suspected cases did not complete all stages of assessment, raising the possibility of attrition bias despite the use of inverse probability weighting. Individuals lost during follow-up may systematically differ in healthcare access, stigma, or seizure severity, potentially influencing both prevalence and treatment-gap estimates. This limitation should be discussed in greater depth.

5. Reliance on self-reported data

Several important variables, including medication adherence, seizure control, treatment effectiveness, and health-seeking behavior, relied primarily on participant self-report. This introduces the possibility of recall bias and social desirability bias, particularly in relation to adherence and perceived seizure outcomes. The limitations associated with self-reported treatment data should therefore be emphasized more explicitly.

6. Interpretation of sociocultural beliefs

The discussion appropriately highlights the importance of sociocultural beliefs in epilepsy care pathways; however, some interpretations risk oversimplifying culturally embedded explanatory models as mere misinformation. Greater engagement with literature on pluralistic healthcare systems and culturally informed health-seeking behaviors would strengthen the discussion and provide a more balanced interpretation of traditional and spiritual beliefs.

7. Limited health-system analysis

Although the manuscript identifies important barriers such as medication cost, drug shortages and limited specialist access, the health-system analysis remains relatively underdeveloped.

Additional discussion of primary healthcare capacity, ASM supply-chain challenges, workforce limitations, and economic barriers would strengthen the policy relevance of the findings.

8. Statistical analysis and reporting

The statistical methods are generally appropriate; however, additional detail regarding handling of clustering, missing data, and potential confounders would improve transparency.

9.References

The reference list is generally appropriate and includes several authoritative sources on epilepsy epidemiology, treatment gaps, and sub-Saharan African epilepsy care. The strong use of regional African literature is a notable strength and provides relevant contextual grounding for the findings.

However, the manuscript would benefit from inclusion of more recent global policy and implementation-science references, particularly WHO mhGAP and IGAP frameworks, task-sharing models, and literature addressing explanatory models of health seeking in epilepsy.

In addition, at least one reference seems irrelevant :

Reference 13:

“Chowdhury et al. Chronic riluzole treatment increases glucose metabolism…”

All the references should be reviewed for relevance and consistency.

10 Recommedations

The recommendations would benefit from stronger linkage to existing implementation frameworks such as WHO mhGAP and IGAP initiatives, as well as evidence from task-sharing epilepsy-care models in similar low-resource settings.

**********

what does this mean?). If published, this will include your full peer review and any attached files.

If you choose “no”, your identity will remain anonymous but your review may still be made public.

**Do you want your identity to be public for this peer review?**  For information about this choice, including consent withdrawal, please see our Privacy Policy

Reviewer #1: **Yes:** Nura Hamidu Alkali

Reviewer #2: No

**********

[NOTE: If reviewer comments were submitted as an attachment file, they will be attached to this email and accessible via the submission site. Please log into your account, locate the manuscript record, and check for the action link "View Attachments". If this link does not appear, there are no attachment files.]

To ensure your figures meet our technical requirements, please review our figure guidelines: https://journals.plos.org/plosone/s/figures

You may also use PLOS’s free figure tool, NAAS, to help you prepare publication quality figures: https://journals.plos.org/plosone/s/figures#loc-tools-for-figure-preparation.

NAAS will assess whether your figures meet our technical requirements by comparing each figure against our figure specifications.

Attachment Submitted filename: PLOS ONE Manus-Review Work.docx

# Author comment: Author response to Decision Letter 1

**DOI:** 10.1371/journal.pone.0355854.r002

3 Jul 2026

PONE-D-26-03687

Barriers to Epilepsy Care: Measuring the Treatment Gap in Rural and Peri-Urban Districts in Ghana

PLOS One

Response to editorial comments

We thank the Editor for these important observations and have revised the manuscript accordingly.

Comment 1: Please ensure that your manuscript meets PLOS ONE's style requirements, including those for file naming.

Response: Thank you. We have revised the manuscript to conform to PLOS ONE formatting requirements, including file naming, manuscript structure, figure presentation, and title page formatting.

Comment 2-5: Journal Requirement - Funding statement, NIHR expansion, and role of funders

Response: Thank you for this observation. We have removed funding-related text from the Acknowledgments section and from the manuscript and updated the Funding Statement accordingly. We have also expanded the acronym “NIHR” as National Institute for Health and Care Research.

We have revised the funding statement: “The UK National Institute for Health and Care Research (NIHR 200134) funded this project through Official Development Assistance (ODA). The views expressed in this publication are those of the authors and not necessarily those of the National Health Service (NHS), the NIHR, or the Department of Health and Social Care”.

Comment 6: Data availability

Response: Thank you. We confirm that the minimal anonymized dataset underlying the findings was prepared and uploaded as Supporting Information files in the initial submission.

Comment 7: Your ethics statement should only appear in the Methods section of your manuscript.

Response: Thank you for this observation. We have revised the manuscript accordingly by moving the ethics statement section to the Methods section of the manuscript and removing it from other sections to ensure consistency with the journal requirements. The necessary changes have been made in the manuscript (Page 12, lines 239-248).

Comment 8: Please ensure that you refer to Figure 1 in your text as, if accepted, production will need this reference to link the reader to the figure.

Response: Thank you for this observation. We have now appropriately cited Figure 1 in the main text of the manuscript to ensure proper linkage between the text and the figure during production.

Comment 9: We note that Figure 1 in your submission contains map images which may be copyrighted.

Response: Thank you for this important observation. The maps presented in Figure 1 were generated by the authors using geographic coordinates collected from the study area and administrative shapefiles obtained from the respective district assemblies (local authority). No copyrighted Google Maps, Google Earth, Street View images, or other proprietary satellite imagery were used in the preparation of the figure. This figure has been published by the same authors elsewhere (1). This has now been properly referenced (Page 7, line 132).

1. Darkwa EK, Asiamah S, Awini E, Sottie C, Godi A, Williams JE, et al. The prevalence and determinants of epilepsy in Ghana: A population‐based study in two districts using a three‐stage approach. Epilepsia. 2026.

Response to Reviewers

We thank the reviewers for their useful comments, and we have revised the manuscript as such

Reviewer # 1

Comment: The authors failed to state in the Methods section that the study was granted ethics committee approval, although the approval letters were shown under "Supporting Information".

Response: Thank you for this comment. The ethics approval statement was included in the original manuscript; however, it was inadvertently placed before the References section. We have now revised the manuscript by moving the ethics statement to the Methods section, where it is more appropriately positioned in line with the journal requirements. The necessary changes have been made in the manuscript (Page 12, lines 239-248).

Comment: Although the statistical analysis was performed appropriately and rigorously, the authors failed to state in the text under "Results" which statistical method they used (eg, Fisher's exact test, chi-square test). They also failed to state some important results in the text and only displayed them on two tables.

Response: Thank you. We have revised the manuscript accordingly by explicitly stating the statistical tests used in the analyses within the Methods section, including the appropriate tests applied for comparisons between groups (Page 11, lines 224-226).

In addition, key findings previously presented only in the tables have now been incorporated into the narrative text of the Results section to improve clarity and interpretation. The necessary changes have been made in the manuscript (Pages 12-13 and 14, lines 256-258, 261-266, 279-281, and lines 298-305).

Reviewer # 2

Comments 1: Treatment gap definition and operationalization

The manuscript defines the epilepsy treatment gap appropriately in conceptual terms; however, its operational definition within the study requires clarification. In practice, treatment status appears to rely largely on reported ASM use in the preceding month, which may not adequately distinguish between untreated epilepsy, undertreatment, interrupted treatment, or poor adherence. Greater clarity regarding how ‘appropriate treatment’ was assessed would strengthen the validity and interpretability of the treatment-gap estimates.

Response: Thank you for this important observation. We have revised the manuscript to provide greater clarity on the operational definition of the epilepsy treatment gap used in this study. Specifically, we have expanded the Methods section to explain how treatment status and ‘appropriate treatment’ were assessed, including the role of recent anti-seizure medication (ASM) use, treatment continuity, and clinical history obtained during the assessment. We have also clarified the limitations associated with relying on self-reported ASM use in the preceding month, particularly regarding the distinction between untreated epilepsy, undertreatment, interrupted treatment, and poor adherence (Pages 10, lines 194-199, Page 20, lines 392-397).

Comment 2: Cross-sectional design and causal interpretation

Several sections of the discussion imply causal relationships between sociocultural beliefs, awareness deficits, and treatment gaps. However, given the cross-sectional observational design, the findings should be interpreted as associations rather than evidence of causality. The discussion would benefit from greater caution, using terms such as ‘associated with’ or ‘may contribute to’ rather than implying direct causal effects.

Response: Thank you. We agree that, given the cross-sectional observational design of this study, causal inferences cannot be established. The Discussion section has been carefully revised to avoid causal language and to ensure that findings are interpreted as associations rather than evidence of direct causality. Terms implying causal relationships, such as “led to,” “resulted in,” or “caused,” have been replaced with more appropriate cautious language such as “was associated with,” “may contribute to,” or “could partly explain” (Pages 17, 18 and 19, lines 322-334 and 350-372).

Comment 3: Diagnostic ascertainment and epilepsy misclassification

The multistage screening design with physician confirmation is a major strength; however, additional details regarding validation of the screening tool would improve methodological rigor. Information on sensitivity, specificity, and diagnostic reliability is lacking. Furthermore, community-based surveys in low-resource settings may underdetect nonconvulsive epilepsies and may overdetect seizure mimics such as syncope or psychogenic nonepileptic events.

Response: Thank you for acknowledging the strength of the multistage screening design. We appreciate this important comment. In response, we have included additional details in the manuscript regarding the validation of the screening tool and provided an appropriate reference to the validation study conducted before this household survey to strengthen the methodological rigor of the paper (Page 8, lines 151-155).

We also acknowledge the potential limitations of community-based epilepsy surveys in low-resource settings, including the possible under detection of nonconvulsive epilepsies and the over-detection of seizure mimics such as syncope or psychogenic nonepileptic events. To minimize these risks, we implemented a multistage screening process, employed trained fieldworkers for standardized data collection, and ensured neurologist confirmation of suspected epilepsy cases at the final stage of assessment. These measures were intended to improve diagnostic accuracy and reduce the likelihood of misclassification. Relevant clarifications have now been incorporated into the Methods and Discussion sections of the revised manuscript (Pages 7 and 20, lines 160-163 and 388-391).

Comment 4: Attrition bias and representativeness

A substantial proportion of suspected cases did not complete all stages of assessment, raising the possibility of attrition bias despite the use of inverse probability weighting. Individuals lost during follow-up may systematically differ in healthcare access, stigma, or seizure severity, potentially influencing both prevalence and treatment-gap estimates. This limitation should be discussed in greater depth.

Response: Thank you. We acknowledge that loss to follow-up across screening stages could have introduced bias and led to underestimation of epilepsy prevalence and treatment gap estimates. To address this, we applied attrition-adjusted prevalence estimation using inverse probability weighting (IPW) to account for participants lost at Stages 2 and 3. Sampling weights were derived from the inverse probability of progression through each screening stage, based on observed characteristics and response patterns. These weights were incorporated into prevalence calculations to reduce attrition bias, improve representativeness of confirmed epilepsy cases, and strengthen the robustness of the estimates. This limitation has now been discussed in greater detail

(Pages 20, lines 380-387).

Comment 5: Reliance on self-reported data

Several important variables, including medication adherence, seizure control, treatment effectiveness, and health-seeking behavior, relied primarily on participant self-report. This introduces the possibility of recall bias and social desirability bias, particularly in relation to adherence and perceived seizure outcomes. The limitations associated with self-reported treatment data should therefore be emphasized more explicitly.

Response: Thank you. We agree that reliance on self-reported data for key variables such as medication adherence, seizure control, treatment effectiveness, and health-seeking behaviour may introduce recall bias and social desirability bias. In response, we have strengthened the Discussion/Limitations section to more explicitly acknowledge these potential sources of bias and their possible influence on the interpretation of treatment-related findings. We now clarify that treatment-related information was primarily based on participant or caregiver self-report, which may have affected the accuracy of reported adherence, seizure outcomes, and care-seeking patterns. These limitations have now been explicitly discussed in the revised manuscript (Page 20, lines 392-397).

Comment 6: Interpretation of sociocultural beliefs

The discussion appropriately highlights the importance of sociocultural beliefs in epilepsy care pathways; however, some interpretations risk oversimplifying culturally embedded explanatory models as mere misinformation. Greater engagement with literature on pluralistic healthcare systems and culturally informed health-seeking behaviors would strengthen the discussion and provide a more balanced interpretation of traditional and spiritual beliefs.

Response: Thank you. We appreciate the reviewer’s emphasis on the need for a more balanced interpretation of sociocultural beliefs within epilepsy care pathways. We agree that traditional and spiritual beliefs should not be framed solely as misinformation, as health-seeking behaviours in many low-resource settings often occur within pluralistic healthcare systems where biomedical, traditional, and spiritual approaches may coexist and shape care pathways. In response, we have revised the Discussion section to adopt a more nuanced interpretation of culturally embedded explanatory models and strengthened the discussion by incorporating relevant literature on pluralistic healthcare systems and culturally informed health-seeking behaviours. (Pages 18, lines 350-363).

Comment 7: Limited health-system analysis

Although the manuscript identifies important barriers such as medication cost, drug shortages and limited specialist access, the health-system analysis remains relatively underdeveloped.

Additional discussion of primary healthcare capacity, ASM supply-chain challenges, workforce limitations, and economic barriers would strengthen the policy relevance of the findings.

Response: Thank you. We agree that expanding the health-system perspective would strengthen the policy relevance of the findings. In response, we have revised the Discussion section to provide a more comprehensive interpretation of the structural and health-system barriers that may influence epilepsy diagnosis, treatment access, and continuity of care. Specifically, we have expanded the discussion to include primary healthcare capacity for epilepsy management, anti-seizure medication (ASM) supply-chain and availability challenges, workforce limitations including restricted access to specialist neurological care. (Page 18, lines 340-349).

Comment 8: Statistical analysis and reporting

The statistical methods are generally appropriate; however, additional detail regarding handling of clustering, missing data, and potential confounders would improve transparency.

Response: Thank you. To account for potential household and community-level clustering inherent in the door-to-door sampling design, cluster-robust standard errors were calculated for all comparative analyses. Potential confounding by age and sex was controlled through exact matching during the case-control selection phase (Page 10 and 11, lines 228-235).

Comment 9: References

The reference list is generally appropriate and includes several authoritative sources on epilepsy epidemiology, treatment gaps, and sub-Saharan African epilepsy care. The strong use of regional African literature is a notable strength and provides relevant contextual grounding for the findings.

However, the manuscript would benefit from inclusion of more recent global policy and implementation-science references, particularly WHO mhGAP and IGAP frameworks, task-sharing models, and literature addressing explanatory models of health seeking in epilepsy.

Response: Thank you for the suggestions. We have revised the manuscript to include more recent global policy and implementation-science references, particularly the WHO Mental Health Gap Action Programme (mhGAP), the Intersectoral Global Action Plan on Epilepsy and Other Neurological Disorders (IGAP 2022–2031), task-sharing approaches, and literature on explanatory models that influences health-seeking behaviour in epilepsy.(Page 18. Lines 344-349).

Comment 10: Recommendations

The recommendations would benefit from stronger linkage to existing implementation frameworks such as WHO mhGAP and IGAP initiatives, as well as evidence from task-sharing epilepsy-care models in similar low-resource settings.

Response: Thank you for this valuable comment. We have strengthened the recommendations section by explicitly linking our findings to existing implementation frameworks, including the WHO Mental Health Gap Action Programme (mhGAP) and the Intersectoral Global Action Plan on Epilepsy and Other Neurological Disorders (IGAP 2022–2031). (Page 21, lines 408-417).

Supplementary Comments (word document)

Comment 1:

1. Under “Methods – Study setting”, lines 114-118 (Page 5):

The authors listed 16 health facilities in Shai-Osudoku District, but only stated “fewer health facilities” for Ningo-Prampram District. There is a need to mention the health facilities in Ningo-Prampra

Attachment Submitted filename: Response _ Reviewers Plos One.pdf

# Peer review: Decision Letter 1

**DOI:** 10.1371/journal.pone.0355854.r003

**Authors:** Hiroki Annaka

21 Jul 2026

Dear Dr. Akpalu,

Thank you for submitting your manuscript to PLOS ONE. After careful consideration, we feel that it has merit but does not fully meet PLOS ONE’s publication criteria as it currently stands. Therefore, we invite you to submit a revised version of the manuscript that addresses the points raised during the review process.

==============================

The reviewer has requested minor revisions. Please make the necessary changes.

==============================

Please submit your revised manuscript by Sep 04 2026 11:59PM. If you will need more time than this to complete your revisions, please reply to this message or contact the journal office at plosone@plos.org. When you're ready to submit your revision, log on to https://www.editorialmanager.com/pone/ and select the 'Submissions Needing Revision' folder to locate your manuscript file.

- A letter that responds to each point raised by the academic editor and reviewer(s). You should upload this letter as a separate file labeled 'Response to Reviewers'.
- A marked-up copy of your manuscript that highlights changes made to the original version. You should upload this as a separate file labeled 'Revised Manuscript with Track Changes'.
- An unmarked version of your revised paper without tracked changes. You should upload this as a separate file labeled 'Manuscript'.

If you would like to make changes to your financial disclosure, please include your updated statement in your cover letter. Guidelines for resubmitting your figure files are available below the reviewer comments at the end of this letter.

If applicable, we recommend that you deposit your laboratory protocols in protocols.io to enhance the reproducibility of your results. Protocols.io assigns your protocol its own identifier (DOI) so that it can be cited independently in the future. For instructions see: https://journals.plos.org/plosone/s/submission-guidelines#loc-laboratory-protocols. Additionally, PLOS ONE offers an option for publishing peer-reviewed Lab Protocol articles, which describe protocols hosted on protocols.io. Read more information on sharing protocols at https://plos.org/protocols?utm_medium=editorial-email&utm_source=authorletters&utm_campaign=protocols.

As the corresponding author, your ORCID iD is verified in the submission system and will appear in the published article. PLOS supports the use of ORCID, and we encourage all coauthors to register for an ORCID iD and use it as well. Please encourage your coauthors to verify their ORCID iD within the submission system before final acceptance, as unverified ORCID iDs will not appear in the published article. *Only*  the individual author can complete the verification step; PLOS staff *cannot*  verify ORCID iDs on behalf of authors.

We look forward to receiving your revised manuscript.

Kind regards,

Hiroki Annaka

Academic Editor

PLOS One

Journal Requirements:

If the reviewer comments include a recommendation to cite specific previously published works, please review and evaluate these publications to determine whether they are relevant and should be cited. There is no requirement to cite these works unless the editor has indicated otherwise.

Please review your reference list to ensure that it is complete and correct. If you have cited papers that have been retracted, please include the rationale for doing so in the manuscript text, or remove these references and replace them with relevant current references. Any changes to the reference list should be mentioned in the rebuttal letter that accompanies your revised manuscript. If you need to cite a retracted article, indicate the article’s retracted status in the References list and also include a citation and full reference for the retraction notice.

[Note: HTML markup is below. Please do not edit.]

Reviewers' comments:

Reviewer's Responses to Questions

**Comments to the Author**

Reviewer #1: All comments have been addressed

Reviewer #2: All comments have been addressed

**********

2. Is the manuscript technically sound, and do the data support the conclusions?

Reviewer #1: Yes

Reviewer #2: Partly

**********

3. Has the statistical analysis been performed appropriately and rigorously?

Reviewer #1: Yes

Reviewer #2: I Don't Know

**********

4. Have the authors made all data underlying the findings in their manuscript fully available?

The PLOS Data policy

Reviewer #1: Yes

Reviewer #2: Yes

**********

5. Is the manuscript presented in an intelligible fashion and written in standard English?

Reviewer #1: Yes

Reviewer #2: Yes

**********

Reviewer #1: Authors have addressed all my earlier comments and suggestions.

1. The authors now stated in the methods section that they had ethical approval for the study.

2. The authors have mentioned the statistical tests used in the study.

3. The authors have now listed the number of facilities in the Ningo-Prampram District

4. The authors have provided the exact start and end dates of the study.

5. The authors have satisfactorily explained the seemingly high attrition rate.

6. The authors have corrected the formatting error at line 193 (page 9).

Reviewer #2: The authors have addressed the majority of the reviewers’ comments and the manuscript has improved substantially. I have only a few remaining comments.

1. Treatment gap definition: Although the operational definition has been clarified, the reported treatment gap reflects recent ASM use rather than treatment adequacy. This distinction should remain explicit when interpreting the findings and comparing them with other studies.

2. Interpretation of findings: The discussion should continue to avoid causal language. Given the cross-sectional design, factors such as lack of awareness, sociocultural beliefs, and reliance on traditional medicine should be described as being associated with, rather than causing, the treatment gap.

**********

what does this mean?). If published, this will include your full peer review and any attached files.

If you choose “no”, your identity will remain anonymous but your review may still be made public.

**Do you want your identity to be public for this peer review?**  For information about this choice, including consent withdrawal, please see our Privacy Policy

Reviewer #1: **Yes:** Nura Hamidu Alkali

Reviewer #2: No

**********

[NOTE: If reviewer comments were submitted as an attachment file, they will be attached to this email and accessible via the submission site. Please log into your account, locate the manuscript record, and check for the action link "View Attachments". If this link does not appear, there are no attachment files.]

To ensure your figures meet our technical requirements, please review our figure guidelines: https://journals.plos.org/plosone/s/figures

You may also use PLOS’s free figure tool, NAAS, to help you prepare publication quality figures: https://journals.plos.org/plosone/s/figures#loc-tools-for-figure-preparation.

NAAS will assess whether your figures meet our technical requirements by comparing each figure against our figure specifications.

# Author comment: Author response to Decision Letter 2

**DOI:** 10.1371/journal.pone.0355854.r004

24 Jul 2026

Manuscript ID: PONE-D-26-03687R1 : Barriers to Epilepsy Care: Measuring the Treatment Gap in Rural and Peri-Urban Districts in Ghana

Response to Reviewers

Dear Hiroki Annaka,

We thank the Academic Editor and reviewers for their evaluation of our revised manuscript and for their feedback. We appreciate that the reviewers found the manuscript to have improved. We have carefully considered the remaining comments and revised the manuscript accordingly. Our reactions are provided below. We have used the opportunity to address minor issues identified in the manuscript in track changes.

Reviewer #1

We thank the reviewer for the positive assessment of our manuscript and appreciate the time and effort invested in reviewing our work.

Reviewer #2

We thank the reviewer for the positive evaluation of our revised manuscript and for the remaining suggestions. We have now addressed each comment.

Comment 1:Treatment gap definition: Although the operational definition has been clarified, the reported treatment gap reflects recent ASM use rather than treatment adequacy. This distinction should remain explicit when interpreting the findings and comparing them with other studies.

Response: Thank you for this observation. We agree that our estimate reflects recent antiseizure medication (ASM) use and should not be interpreted as a measure of treatment adequacy. We have revised the Discussion to explicitly state that the treatment gap reported in this study is based on recent ASM use reflecting lack of current treatment rather than the overall adequacy or quality of epilepsy management. We have also emphasized this distinction when comparing our findings with previous studies that may have used different operational definitions of the treatment gap. (Page 16, lines 301-310)

Comment 2: Interpretation of findings: The discussion should continue to avoid causal language. Given the cross-sectional design, factors such as lack of awareness, sociocultural beliefs, and reliance on traditional medicine should be described as being associated with, rather than causing, the treatment gap.

Response: We appreciate this comment. We have carefully reviewed the Discussion and replaced any remaining causal wording with language appropriate for a cross-sectional study. Throughout the manuscript, statements implying causation have been revised to describe these factors as being associated with the treatment gap rather than causing it. This ensures that the interpretation of our findings accurately reflects the observational nature of the study. (Page 16-19, line 300-367).

Sincerely,

Prof. Albert Akpalu, on behalf of the author team

Attachment Submitted filename: Response to Reviewers 24 July 2026.pdf

# Editor report: Decision Letter 2

**DOI:** 10.1371/journal.pone.0355854.r005

**Authors:** Hiroki Annaka

27 Jul 2026

Barriers to Epilepsy Care: Measuring the Treatment Gap in Rural and Peri-Urban Districts in Ghana

PONE-D-26-03687R2

Dear Dr. Akpalu,

We’re pleased to inform you that your manuscript has been judged scientifically suitable for publication and will be formally accepted for publication once it meets all outstanding technical requirements.

Within one week, you’ll receive an e-mail detailing the required amendments. When these have been addressed, you’ll receive a formal acceptance letter and your manuscript will be scheduled for publication.

An invoice will be generated when your article is formally accepted. Please note, if your institution has a publishing partnership with PLOS and your article meets the relevant criteria, all or part of your publication costs will be covered. Please make sure your user information is up-to-date by logging into Editorial Manager at Editorial Manager® and clicking the ‘Update My Information' link at the top of the page. For questions related to billing, please contact billing support.

If your institution or institutions have a press office, please notify them about your upcoming paper to help maximize its impact. If they’ll be preparing press materials, please inform our press team as soon as possible -- no later than 48 hours after receiving the formal acceptance. Your manuscript will remain under strict press embargo until 2 pm Eastern Time on the date of publication. For more information, please contact onepress@plos.org.

Kind regards,

Hiroki Annaka

Academic Editor

PLOS One

Additional Editor Comments (optional):

Reviewers' comments:

# Editor report: Acceptance letter

**DOI:** 10.1371/journal.pone.0355854.r006

**Authors:** Hiroki Annaka

PONE-D-26-03687R2

PLOS One

Dear Dr. Akpalu,

I'm pleased to inform you that your manuscript has been deemed suitable for publication in PLOS One. Congratulations! Your manuscript is now being handed over to our production team.

At this stage, our production department will prepare your paper for publication. This includes ensuring the following:

* All references, tables, and figures are properly cited

* All relevant supporting information is included in the manuscript submission,

* There are no issues that prevent the paper from being properly typeset

You will receive further instructions from the production team, including instructions on how to review your proof when it is ready. Please keep in mind that we are working through a large volume of accepted articles, so please give us a few days to review your paper and let you know the next and final steps.

Lastly, if your institution or institutions have a press office, please let them know about your upcoming paper now to help maximize its impact. If they'll be preparing press materials, please inform our press team within the next 48 hours. Your manuscript will remain under strict press embargo until 2 pm Eastern Time on the date of publication. For more information, please contact onepress@plos.org.

You will receive an invoice from PLOS for your publication fee after your manuscript has reached the completed accept phase. If you receive an email requesting payment before acceptance or for any other service, this may be a phishing scheme. Learn how to identify phishing emails and protect your accounts at https://explore.plos.org/phishing.

If we can help with anything else, please email us at customercare@plos.org.

Thank you for submitting your work to PLOS ONE and supporting open access.

Kind regards,

PLOS ONE Editorial Office Staff

on behalf of

Dr. Hiroki Annaka

Academic Editor

PLOS One
